# Supplementary material for: Clinical Features and Outcomes of Conversion Therapy in Patients with Unresectable Hepatocellular Carcinoma
Source: Cancers (Basel). 2023 Oct 30;15(21):5221. doi: 10.3390/cancers15215221 (PMC10650115; doi:10.3390/cancers15215221)
Supplement: Supplementary file 1 [file cancers-15-05221-s001.zip › Cancers_Supplementary_Table_1.pdf]

Table S1. Response to treatment with lenvatinib and atezolizumab plus bevacizumab for hepatocellular carcinoma.

**Evaluation (RECIST version 1.1)**	**Atezo+Bev** ***n (%)*** ***(n=113)***	**LEN** ***n (%)*** ***(n=131)***
Complete response	1 (0.8)	2 (1.5)
Partial response	22 (19.5)	33 (25.1)
Stable disease	67 (59.3)	82 (62.6)
Progressive disease	23 (20.4)	14 (10.7)
Objective response rate (%)	20.4	26.7*
Disease control rate (%)	79.6	89.3

LEN, lenvatinib; RECIST, Response Evaluation Criteria in Solid Tumors
