# Supplementary material for: Clinical Features and Outcomes of Conversion Therapy in Patients with Unresectable Hepatocellular Carcinoma
Source: Cancers (Basel). 2023 Oct 30;15(21):5221. doi: 10.3390/cancers15215221 (PMC10650115; doi:10.3390/cancers15215221)
Supplement: Supplementary file 1 [file cancers-15-05221-s001.zip › Cancers_Supplementary_Table_2.pdf]

Table S2. Baseline patient characteristics of conversion cases.

	Patient characteristics at baseline										
Case	Drugs	Age	Sex	Etiology	Treatment line	AFP (ng/mL)	Child-Pugh score	mALBI score	Tumor number	Maximum tumor size (mm)	BCLC stage
1	LEN	74	M	NASH	First	13	5	2a	5	23	B
2	LEN	72	M	Alcohol	First	6	5	1	4	51	B
3	LEN	69	M	Alcohol	First	1,637	5	2a	3	73	B
4	LEN	71	F	NASH	First	4	5	2a	10	61	B
5	LEN	79	M	HCV	First	10,950	6	1	2	42	B
6	LEN	71	F	NASH	First	11	6	2b	1	64	B
7	A/B	80	M	HCV	First	162	5	2a	1	81	C
8	A/B	76	F	HCV	First	832	5	2a	5	33	B
9	A/B	60	M	NASH	Third	3,024	6	2a	3	32	B
10	A/B	78	M	HCV	First	5	5	2a	6	28	B
11	A/B	78	F	HCV	First	268	5	1	4	21	B
12	A/B	79	M	Alcohol	First	3	5	1	5	68	B
Median	-	75	-	-	-	88	5	-	4	47	-

AFP, alpha-fetoprotein; ALBI, albumin–bilirubin; BCLC, Barcelona Clinic Liver Cancer; LEN, Lenvatinib; HBV, hepatitis B virus; HCV, hepatitis C virus
