# Supplementary material for: Clinical Features and Outcomes of Conversion Therapy in Patients with Unresectable Hepatocellular Carcinoma
Source: Cancers (Basel). 2023 Oct 30;15(21):5221. doi: 10.3390/cancers15215221 (PMC10650115; doi:10.3390/cancers15215221)
Supplement: Supplementary file 1 [file cancers-15-05221-s001.zip › Cancers_Supplementary_Table_3.pdf]

Table S3. Characteristics of patients with unresectable hepatocellular carcinoma treated with and without conversion therapy in propensity score matching cohort.

**Characteristics**	**All** (***n***=**20**)	**Conversion +** (***n***=**10**)	**Conversion -** (***n***=**10**)	***p*-value**
Age, median [Quartiles], (years)	72 [68–78]	73 [71–78]	69 [65–77]	0.35
Sex (male/female), *n*	15/5	7/3	8/2	1
ECOG-PS (0/1), *n*	18/2	8/2	10/0	0.47
Etiology (HBV/HCV/NBNC), n	3/6/11	0/4/6	3/2/5	0.16
Platelets, median [Quartiles], (10^4/μL)	16.1 [11.7–20.6]	18.8 [14.2–21.0]	12.6 [10.9–18.9]	0.36
M2BpGi [Quartiles] (C.O.I)	1.11 [0.97–2.07]	1.09 [0.97–1.41]	1.61 [0.83–2.18]	0.80
Child–Pugh score (5/6), *n*	17/3	8/2	9/1	1
mALBI Grade (1/2a), *n*	8/12	4/6	4/6	1
Portal vein invasion (absent/present), *n*	18/2	9/1	9/1	1
Extrahepatic spread (absent/present), *n*	19/1	10/0	9/1	1
AFP, median [Quartiles] (ng/ml)	19 [5–176]	88 [8–256]	19 [6.5–36]	0.26
BCLC stage (B/C), *n*	17/3	9/1	8/2	1
Drug therapy (Lenvatinib/Atezolizumab+ Bevacizumab)	12/8	6/4	6/4	1
Treatment line (first line/later line), n	19/1	10/0	9/1	1

AFP, alpha-fetoprotein; ALBI, albumin–bilirubin; BCLC, Barcelona Clinic Liver Cancer; ECOG-PS, Eastern Cooperative Oncology Group performance status; HBV, hepatitis B virus; HCV, hepatitis C virus; LEN, lenvatinib; M2BPGi mac-2 binding protein glycosylation isomer; NBNC, non-B non-C; TAE/TACE, transcatheter embolization/chemoembolization
