# Supplementary material for: Clinical Features and Outcomes of Conversion Therapy in Patients with Unresectable Hepatocellular Carcinoma
Source: Cancers (Basel). 2023 Oct 30;15(21):5221. doi: 10.3390/cancers15215221 (PMC10650115; doi:10.3390/cancers15215221)
Supplement: Supplementary file 1 [file cancers-15-05221-s001.zip › Cancers_Supplementary_Table_4.pdf]

Table S4. Univariate analyses of factors that influenced Atezolizumab + Bevacizumab conversion therapy.

Variables	Category	No. of Patients (%)		Univariate
		Atezo+Bev Conversion +	Atezo+Bev Conversion -	p-value
Age, (years)	≥75	4 (67)	49 (48)	0.42
	<75	2 (33)	58 (52)	
Sex	Male	4 (67)	93 (87)	0.20
	Female	2 (33)	14 (13)	
Etiology	Viral	4 (67)	52 (52)	0.10
	No-viral	2 (33)	55 (48)	
ECOG PS	1	1 (17)	24 (22)	1
	0	5 (83)	83 (85)	
mALBI Grade 1+2a	Yes	6 (100)	58 (54)	0.03
	No	0 (0)	49 (46)	
Child-Pugh score 5	Yes	1 (17)	39 (36)	0.42
	No	5 (83)	68 (64)	
BCLC stage	B	5 (83)	60 (56)	0.16
	C	1 (17)	57 (44)	
AFP level (ng/mL)	≥400	1 (17)	38 (36)	0.66
	<400	5 (83)	69 (64)	
Treatment line	First line	5 (83)	65 (61)	0.41
	Later line	1 (17)	42 (39)	
