# Supplementary figures and images for: Clinical Features and Outcomes of Conversion Therapy in Patients with Unresectable Hepatocellular Carcinoma
Source: Cancers (Basel). 2023 Oct 30;15(21):5221. doi: 10.3390/cancers15215221 (PMC10650115; doi:10.3390/cancers15215221)

Supplementary Fig.1

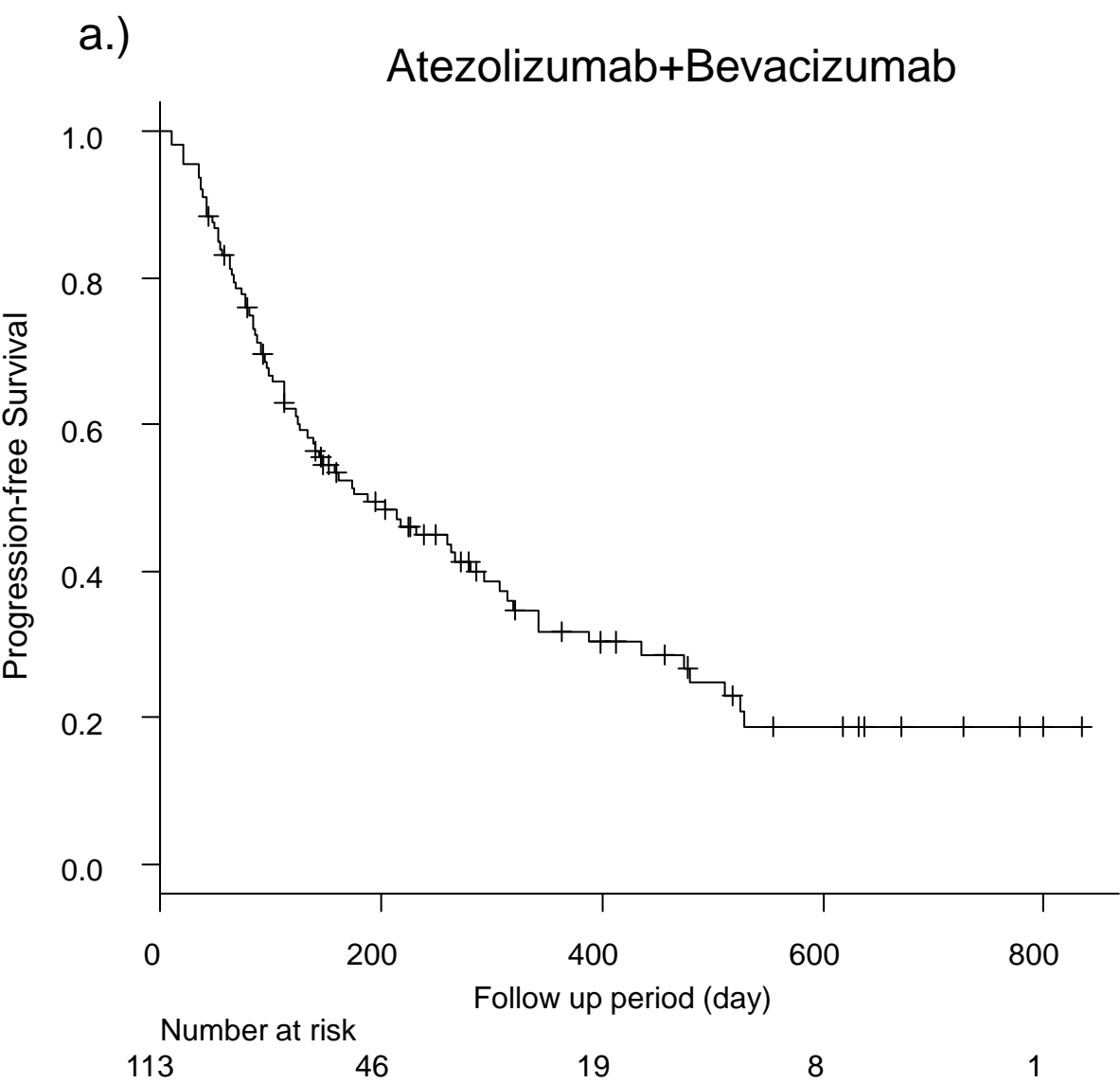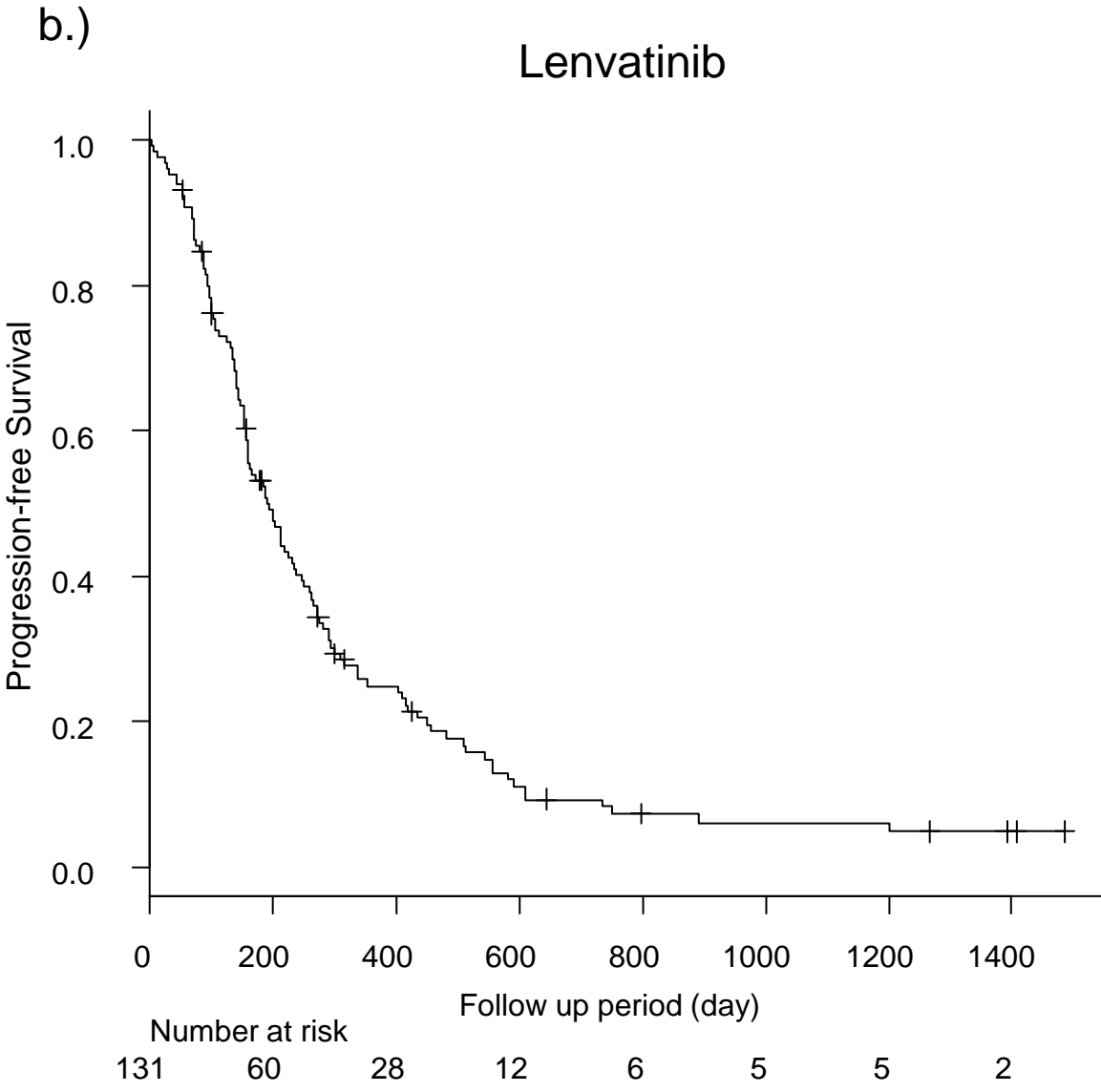

Supplement: Supplementary file 1 [file cancers-15-05221-s001.zip › Supple 1 Ver2_Cancers_Conversion_Figure_.pdf]

Supplementary Fig.2

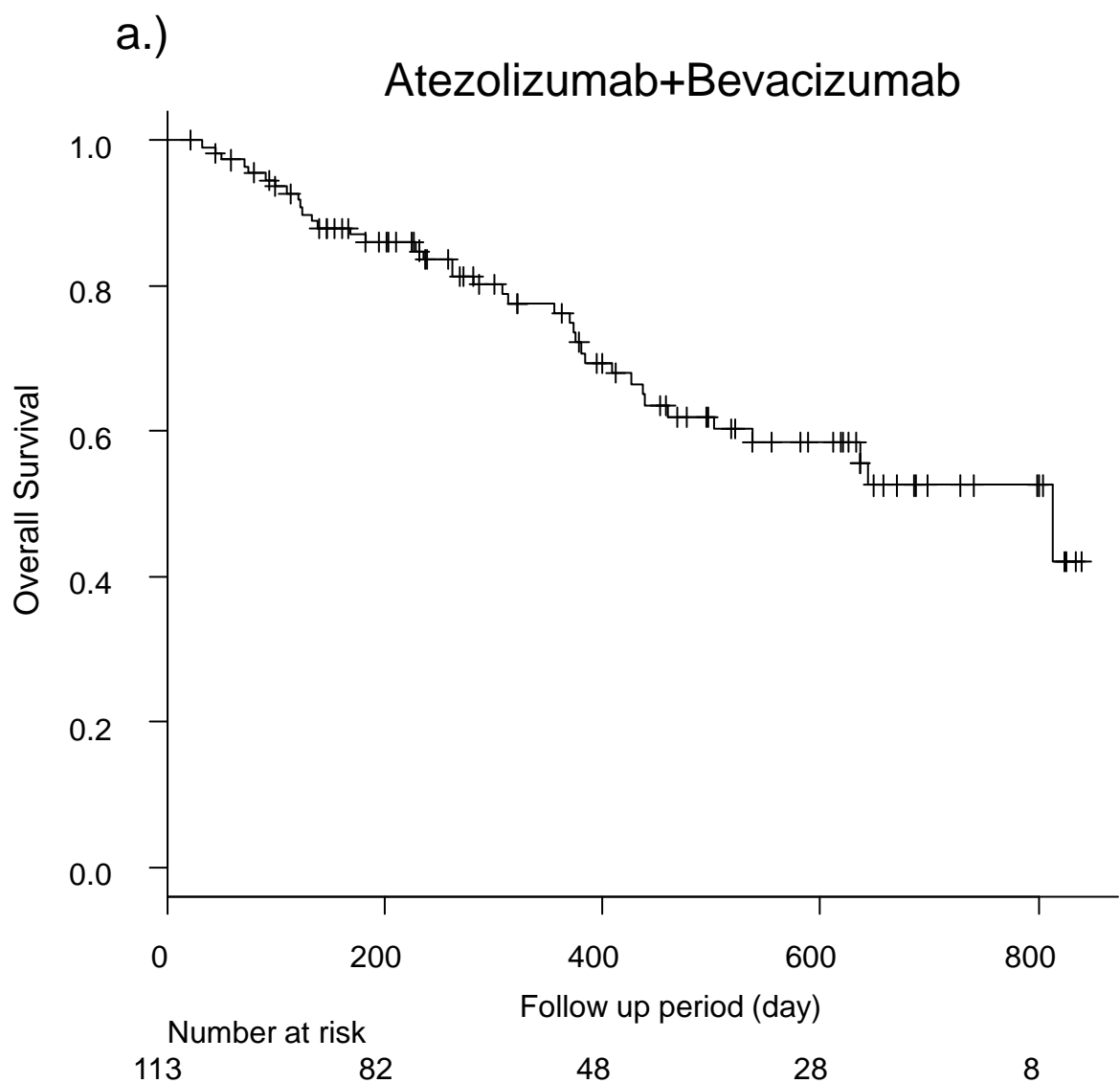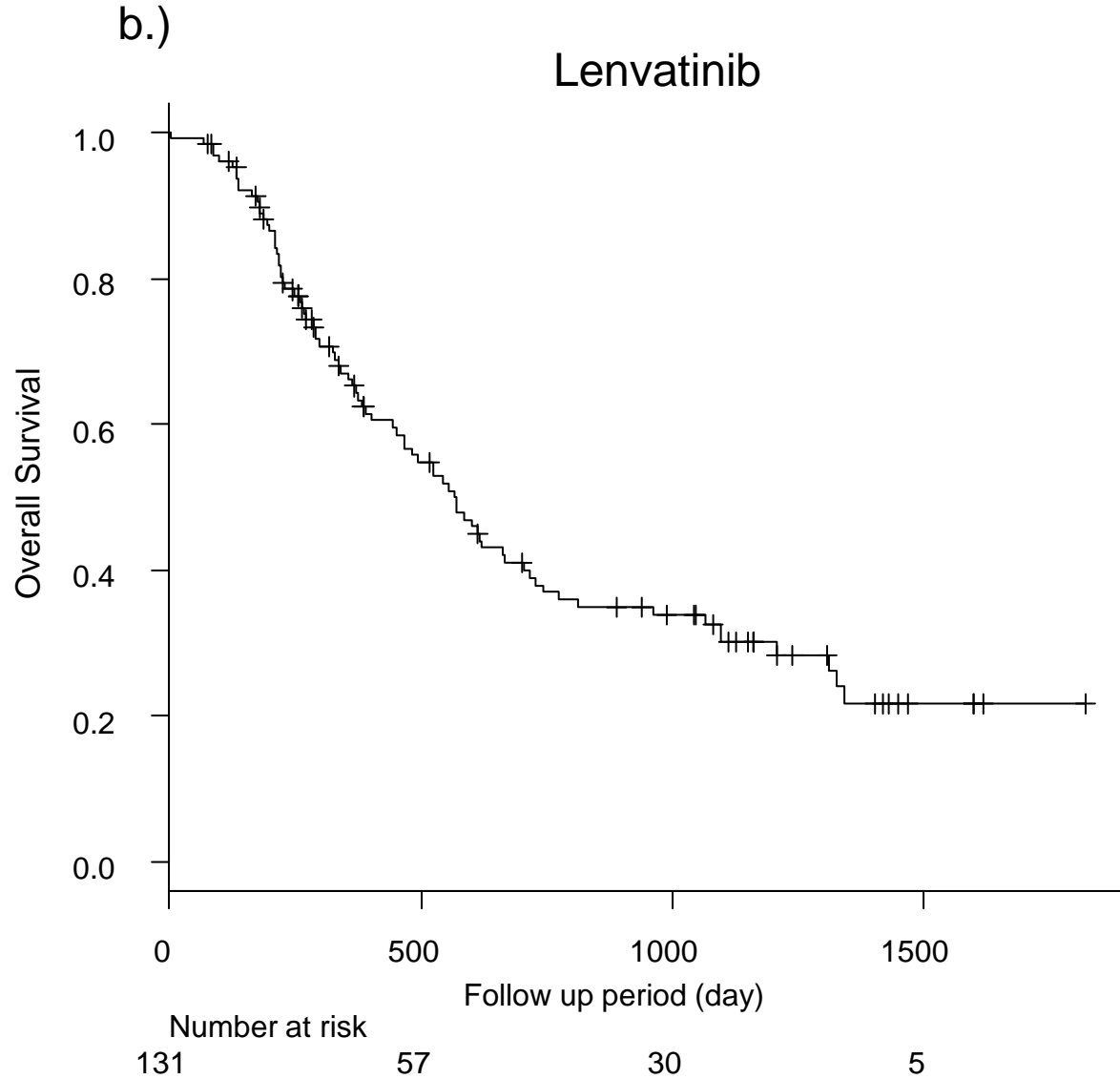

Supplement: Supplementary file 1 [file cancers-15-05221-s001.zip › Supple 2 ver2_Cancers_Conversion_.pdf]

Supplementary Fig.3

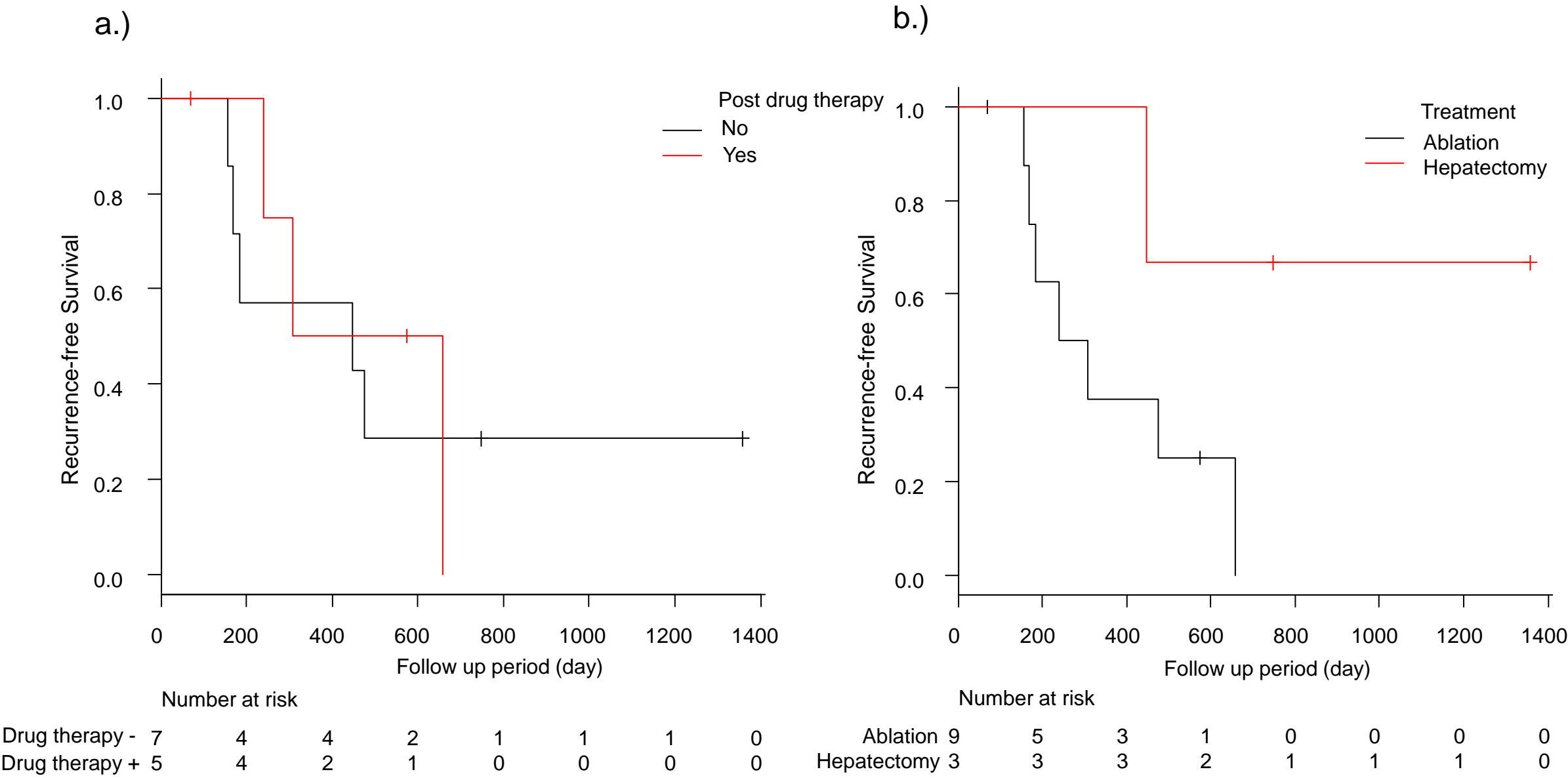

Supplement: Supplementary file 1 [file cancers-15-05221-s001.zip › Supple 3_Cancers_Conversion_Figure_.pdf]

Supplementary Fig.4

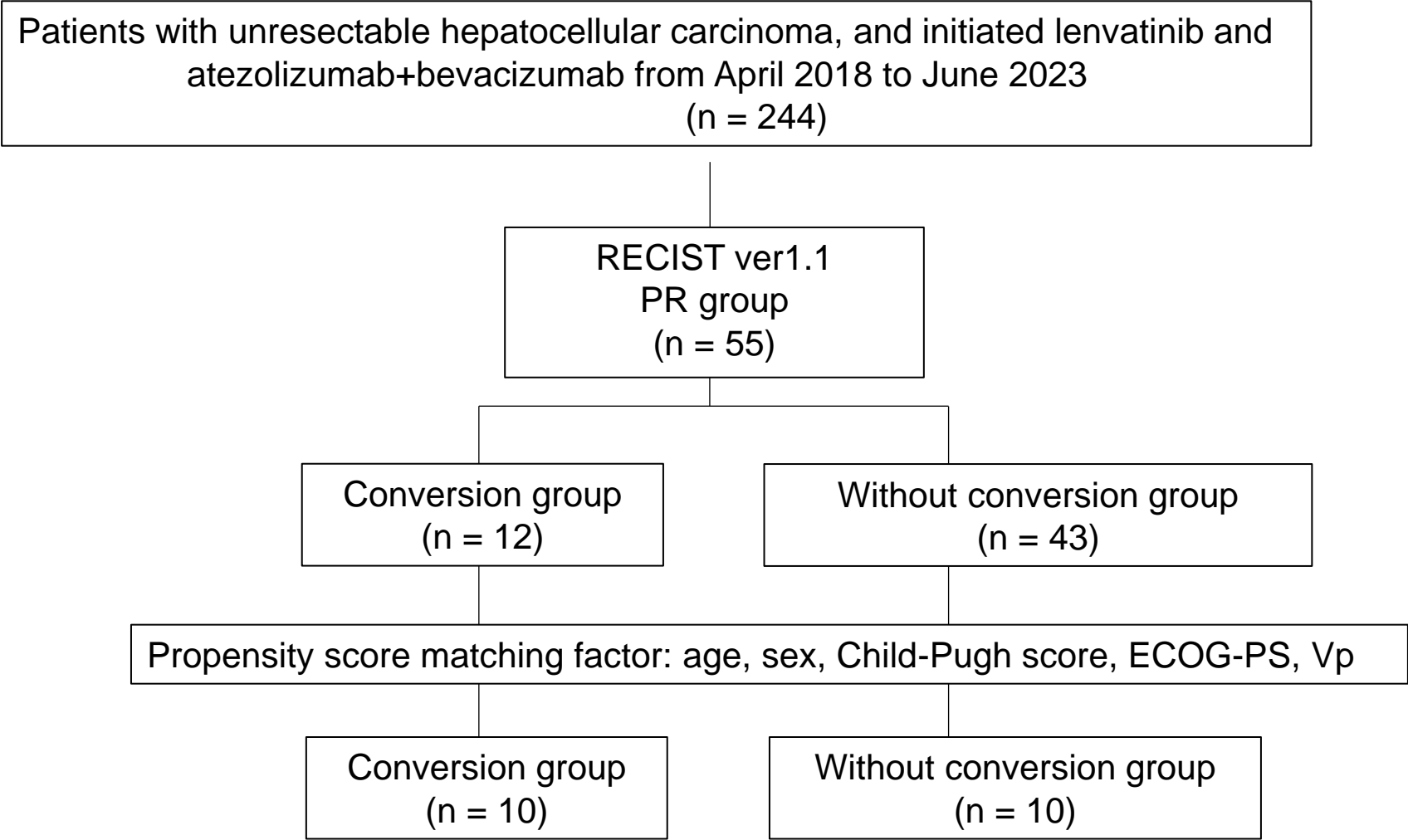

Supplement: Supplementary file 1 [file cancers-15-05221-s001.zip › Supple 4_Cancers_Conversion_.pdf]
